# Characteristics of Human-Like Virtual Profiles in Relation to Audience Reach and Engagement on Instagram: Secondary Data Analysis

**DOI:** 10.2196/86233

**Published:** 2026-06-05

**Authors:** Alexandra Maria Lebrecht, Winze Tam, Omar Merlo, Andreas Benedikt Eisingerich

**Affiliations:** 1 Imperial Business School, Imperial College London, Exhibition Road, South Kensington Campus, London, SW7 2AZ, United Kingdom, 44 20 7589 511

**Keywords:** virtual influencers, artificial intelligence, generative AI, Instagram, audience engagement, audience reach, photorealism, content analysis, ethical design, computer-generated image

## Abstract

**Background:**

Prior research on human-like virtual profiles (VPs)—computer-generated imagery (CGI; displaying legible artificiality) or artificial intelligence (AI)–generated personas on social media—is often based on small samples and limited data. Furthermore, prior studies group highly photorealistic virtual influencers with abstract virtual characters, complicating comparisons. Recent generative AI has enabled large-scale production of synthetic content. These advances have drastically increased the quality and volume of synthetic media. Much of the prior literature predates these developments, leaving open questions about how posting behavior and VP design now relate to reach and engagement outcomes.

**Objective:**

This study aimed to examine how posting behavior and key VP attributes, namely, photorealism; physical, behavioral, and narrative consistency; and human copresence related to reach and engagement across content formats (static images vs videos) on Instagram.

**Methods:**

A total of 157 human-like female VPs were included in the final Instagram dataset. During the initial screening stage, 5 human-like male macro and mega VPs were identified but excluded because their number was too small to support meaningful subgroup comparison. Engagement was operationalized as like rate (likes or followers) for images and videos; reach was measured via examined absolute impressions. Performance was summarized using the single best-performing post and the arithmetic mean of the top 3 posts per format. Profiles and posts were further coded using predefined content variables covering visual realism, identity consistency, copresence structure, appearance patterns, and body-type representation.

**Results:**

Higher-performing content was more commonly observed among larger profiles, particularly for video metrics. Among videos exceeding 20 million impressions, out of 12 videos, 7 (58%) were produced by CGI-like VPs, whereas high video like rates were more often observed among photorealistic or face-swapped profiles. In the image engagement analysis, out of 8 highest-like-rate posts, 6 (75%) featured VPs with dark hair and dark eyes, and out of the 8 posts, 4 (50%) included copresence of multiple subjects. Furthermore, of the 157 top-performing profiles reviewed, 122 (77.71%) demonstrated stable visual identity and consistent behavioral and narrative presentation, whereas noticeable inconsistencies were rarely observed in this subset. These findings represent descriptive patterns within established macro- and mega-level profiles rather than as causal predictors of growth.

**Conclusions:**

Among established human-like female VPs on Instagram, higher engagement and reach frequently co-occurred with larger profile scale, identity coherence, and transparent virtual presentation than with photorealism alone. CGI-like aesthetics aligned more with algorithmic distribution, whereas photorealistic motion content featured more prominently among posts with high active engagement. These results suggest that audiences may respond more favorably to coherent and legible virtual identities than to ambiguous realism. Because the study was restricted to macro- and mega-level profiles, the observed traits should be interpreted as characteristics of successful incumbents rather than as general predictors of VP growth.

## Introduction

### Overview

A virtual human or virtual profile (VP) is a fictional character on social media, operated by a creative professional or agency, that defines the full spectrum of its online presence, including physical appearance, personality, and narrative identity [1]. When VP 2, one of the earliest and most prominent digital-born human-like virtual celebrities, debuted on Instagram in 2016, the idea of a convincingly human-like synthetic persona was still largely unfamiliar to mainstream audiences [2]. Since then, human-like VPs have moved from novelty to a visible creator category, supported by rapid improvements in generative artificial intelligence (AI) that have lowered production barriers and increased the visual quality and volume of synthetic content distributed on major platforms [3,4].

This technological shift matters for research because much of the previous virtual influencer (VI) literature was produced under materially different conditions. Earlier work often evaluated relatively small samples, relied on manual selection of posts, or aggregated fundamentally diverse character styles, including abstract non–human-like VPs into a single “VI” category [5,6]. In practice, however, even the human-like VP category occupies a spectrum ranging from explicitly computer-generated imagery (CGI; displaying legible artificiality)–like personas to highly photorealistic VPs. These distinctions are not merely aesthetic. They may influence how audiences interpret the account’s intent (entertainment vs deception), how authenticity is inferred, and how platform governance (eg, disclosure expectations or AI-content labels) interacts with user response [7-9].

Against this backdrop, this study examines reach and engagement performance among human-like female VPs on Instagram using a contemporary secondary dataset. The study is motivated by 2 guiding principles of today’s VP environment. First, video is increasingly central to platform distribution and creator strategy, and it offers impressions and reach information that is not systematically visible for static images on Instagram; consequently, studies that treat image-based engagement as representative of overall performance may understate the role of algorithmic amplification [10]. Second, audience literacy has changed: as users have become more familiar with synthetic media, the success conditions for VPs may have shifted from maximizing photorealism toward maximizing identity coherence, perceived transparency, and authenticity [7,8].

In line with these developments, the study evaluates 3 core design characteristics, namely, photorealism, physical and behavioral consistency, and copresence structure, and how they descriptively relate to reach and engagement across content formats. Accordingly, the following exploratory propositions were specified: (P1) Photorealism and explicit CGI-likeness co-occur with distinct reach and engagement patterns, rather than one style uniformly outperforming the other. (P2) Stable physical, ethnic, and behavioral presentation is frequently observed among high-reach and high-engagement profiles. (P3) Posts featuring copresence, whether human-VP or VP-VP, are frequently observed among high-performing posts, although solo VP posts also appear in this subset. (P4) Specific appearance patterns, such as dark hair, dark eyes, and exaggerated body proportions, recur among top-performing VPs.

By examining these exploratory propositions, this study aims to contribute to the growing body of research on human-AI interaction, shedding light on how audiences engage emotionally and behaviorally with AI-generated personas in digital environments. The study further advances knowledge by clarifying which VP content deployment strategies and design signals are most prominent among high-performing accounts on Instagram. It also strives to provide a more valid basis for comparison than approaches that rely primarily on high-performing outliers or noncomparable metrics [5,6,11].

### Background

Prior research examining the degree of human-likeness in VIs and its association with user engagement has yielded mixed results. While some studies suggest that VIs can elicit lower perceived authenticity and trustworthiness than physical human influencers in certain contexts [7,12], other work indicates that engagement outcomes can be comparable in some settings [10] and may be shaped by novelty, curiosity, and creator control [6]. This divergence is consistent with findings in related fields such as embodied and conversational agents and digital health, where human-like cues can strengthen social presence and relational engagement but, consistent with the “uncanny valley” hypothesis, may also reduce affinity when high realism creates expectancy violations or perceptual discomfort [13-15]. Achieving the right balance of human-likeness is therefore crucial for maintaining trust, perceived authenticity, and engagement in human-virtual interactions [13].

Several studies have compared VIs with traditional human influencers to better understand their relative effectiveness, advantages, and limitations. These analyses have produced mixed results. Some research suggests that VIs may be perceived as less trustworthy or relatable than human influencers but benefit from novelty, originality, and design control [6,16]. Conversely, other studies emphasize that VIs often struggle to replicate the emotional connection and sense of authenticity that human influencers achieve with their audiences [12,17]. This phenomenon can be explained through social identity theory, which posits that individuals are more likely to identify with others who display similar human or social characteristics [18].

Authenticity, a central construct in influencer marketing and digital communication, has also been explored extensively in relation to VPs [7,8]. Prior work highlights that a VP’s success depends not only on visual or behavioral realism but also on the consistency of these authentic cues across contexts [19]. More recent studies identify clusters of VI attributes that foster user compliance, identification, and internalization, and are linked to specific behavioral responses [20]. Together, these findings suggest that perceived authenticity and relational consistency are fundamental to sustaining engagement in human-AI interactions. Furthermore, navigating authenticity remains a primary challenge in influencer marketing, as creators and brands must continuously negotiate trust with their audiences [21]; a dynamic that becomes even more complex when the influencer is entirely synthetic.

Insights regarding digital health show that relational and safety factors also shape human-virtual interactions, offering perspectives that complement research on VPs. In digital health, for instance, scholars have examined embodied conversational agents in clinical psychology and related areas. Embodied conversational agents are used to deliver automated human-support factors and are increasingly explored for psychotherapeutic interventions; however, controlled evidence of clinical effectiveness remains scarce [14]. This idea aligns with the notion of relational agents, computational systems intentionally designed to build and sustain long-term social-emotional relationships with users to facilitate ongoing engagement [15].

However, research in health communication also reveals the risks of deploying conversational AI without sufficient safeguards. An observational study of Siri, Alexa, and Google Assistant identified potential patient safety risks, as these systems occasionally provided incomplete or unsafe responses to medical queries [22]. Similarly, a systematic review of mental health chatbots found only 2 studies assessing safety, both reporting no adverse events yet highlighting the low overall quality of evidence [23]. More recent work on AI-based conversational agents for chronic conditions has reported generally positive results for usability, satisfaction, and engagement, although variability remains high and standardized evaluation is lacking [24]. A 1-year observational study of a chatbot for patients with breast cancer likewise found high engagement and satisfaction but emphasized the need for controlled trials to assess health outcomes [25]. Across these studies, design quality, authenticity, and relational engagement consistently emerge as key factors associated with user trust and sustained use.

Together, these streams of research demonstrate that the perceived authenticity and relational design of digital agents—whether health chatbots or VIs—are closely related to human engagement and trust. Yet, despite these parallels, the factors relating to engagement among highly photorealistic, AI-generated VPs remain underexplored. Many existing studies were conducted before the emergence of sophisticated generative AI tools capable of creating lifelike images and videos. Consequently, prior findings may not reflect current user perceptions and behaviors toward hyperrealistic virtual personas. This study addresses this gap by analyzing contemporary VPs created using advanced generative AI technologies to better understand how photorealism, behavioral consistency, and contextual alignment relate to user engagement and trust in digital environments.

## Methods

### Study Design

This research investigates how VP design and posting outcomes relate to 2 distinct performance concepts: interaction-based engagement and distribution-based reach. More specifically, engagement is operationalized as like rate (likes divided by follower count) for both images and videos, reflecting an observable, comparable interaction signal. Furthermore, reach is examined only for video content via absolute impressions, with exceptional virality for the category of human-like VPs defined as videos exceeding 20 million impressions, a threshold that captures rare, high-magnitude distribution events without normalizing by follower count. This distinction is conceptually important: impressions reflect algorithmic exposure enabled by positive feedback loops, whereas *likes* reflect active user response, and the 2 may not move in parallel across VP types or content strategies [6,10]. Because inferential statistical comparison was not feasible to be performed on the underlying dataset, the study’s conclusions are limited to descriptive patterns and exploratory interpretation.

### Virtual Content Design Coding

#### Overview

A structured content-coding procedure was applied across the full sample of 157 human-like female VPs included in the final dataset. Coding focused on stable profile-level attributes and recurring content design features that could plausibly relate to audience response. All coding variables were defined in advance and applied using fixed operational decision rules to improve transparency and reduce subjectivity. The complete coding framework, including operational definitions and decision rules, is provided in Table S1 in Multimedia Appendix 1. This full-sample coding provides the baseline descriptive distribution used to contextualize whether specific traits observed among high-performing outliers represent a descriptive concentration relative to the broader VP ecosystem. Following this full-sample profile-level coding, a more detailed postlevel structured review was conducted only for posts meeting the predefined performance thresholds specified in the research design.

#### Core Design Variables

Consistent with the study’s exploratory propositions, the primary appearance-related differentiation concerned the visual realism of the VP. Each profile was coded as either CGI-like or photorealistic. Profiles were classified as CGI-like when their artificial nature was visually explicit through stylized textures, nonphotographic rendering, exaggerated proportions, or clearly synthetic facial features. Profiles were classified as photorealistic when visual cues closely resembled human photographic realism and could plausibly be mistaken for real human imagery, particularly in static image content.

In addition, profiles were coded for identity and content consistency using predefined observational criteria. Consistency was coded dichotomously (consistent vs inconsistent) based on the stability of facial identity, body representation, overall visual appearance, and behavioral or narrative presentation across posts. Profiles showing repeated and noticeable shifts in these features were coded as inconsistent. That is, minor variation attributable to pose, clothing, lighting, or camera angle was not sufficient for an inconsistency code unless it materially altered the apparent identity of the VP.

#### Copresence Coding

To assess the social structure of depicted content, each analyzed post was coded for copresence structure using mutually exclusive visual categories: (1) solo VP content, in which 1 focal VP appeared without any additional clearly identifiable actor; (2) VP-VP copresence, in which 2 or more VPs appeared together within the same frame; (3) VP-human copresence, in which a VP appeared alongside a clearly identifiable human individual; and (4) no codable copresence, in which visual material was insufficient to classify additional actors with confidence.

Because hybrid face-swapped content may otherwise conflate narrative structure with production technique, face-swapping was treated separately as a production-related characteristic rather than as a copresence category in itself. Accordingly, a face-swapped video showing only 1 apparent actor was coded as solo VP content, whereas a face-swapped video showing a VP alongside another clearly identifiable human was coded as VP-human copresence.

#### Exploratory Appearance Patterns

An additional exploratory coding layer was introduced following the observation of recurring visual patterns among high-performing profiles. Specifically, hair and eye color combinations were coded to examine whether certain appearance traits appeared repeatedly among the top-performing posts. Four categories were used: (1) dark hair and dark eyes, (2) dark hair and bright eyes, (3) bright hair and dark eyes, and (4) bright hair and bright eyes.

These appearance categories were treated as descriptive variables only and were not intended to imply fixed biological or cultural preference structures.

#### Body-Type Coding

Finally, profiles were coded for body-type representation using a binary distinction between exaggerated sexual dimorphism and nonexaggerated body representation. This variable was operationalized using observable visual cues, including markedly amplified bust-to-waist and/or hip-to-waist contrast relative to the surrounding body proportions depicted in the account’s content. Because the final analytic sample included only human-like female VPs, this coding variable was developed for female-presenting bodies and should not be interpreted as a gender-neutral anthropometric classification. The variable was included as a descriptive coding dimension to capture recurring stylization patterns in the sample VP ecosystem rather than to assign normative judgments about body shape.

Because generative AI and CGI frequently produce hyperstylized anatomies that need not conform to human biological norms, applying standard clinical anthropometric thresholds (eg, World Health Organization waist-to-hip ratios) is not methodologically appropriate for synthetic media. Instead, our operational definition of exaggerated sexual dimorphism is grounded in media studies literature on the sexualization and unrealistic body representation of female characters in virtual environments and video games [26]. This approach ensures that the coding captures virtual hyperidealization rather than treating synthetic bodies as human medical cases.

#### Coding Procedure

All coding was conducted using predefined operational definitions and decision rules to minimize subjectivity. Ambiguous cases—such as unclear background figures, uncertain content provenance, or insufficient visual information—were excluded from variable-specific coding rather than resolved inferentially. The coded variables were subsequently used in descriptive pattern analysis.

VP design content was coded by the first author using a predefined coding framework. To assess reliability, a second trained coder independently coded a random subsample comprising 20% of the dataset. Intercoder reliability was substantial (Cohen κ=0.76), indicating good reproducibility of the coding approach.

### Research Design

**Figure 1.**
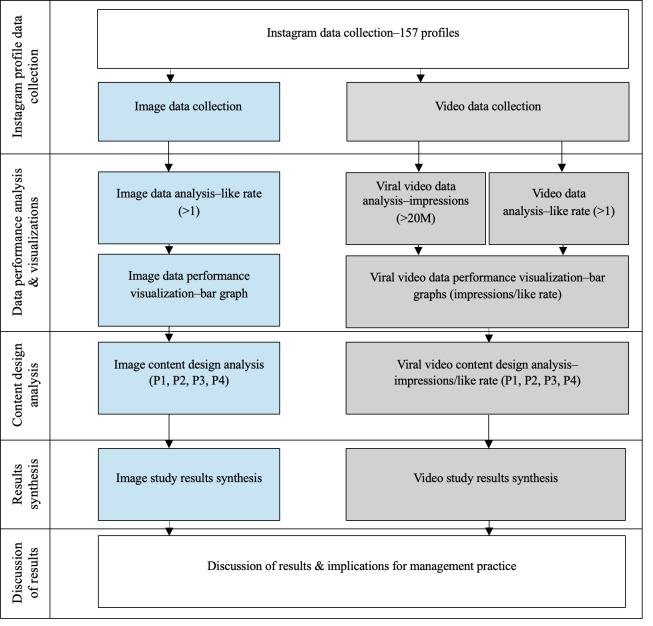
Research design flowchart.

This study used a multiphase research design to systematically examine the relationships between virtual content formats (images and videos) and audience engagement patterns on Instagram. The design integrates quantitative engagement metrics with qualitative profile and content evaluations to better understand how design characteristics relate to user interaction with human-like VPs. Figure 1 provides an overview of the research process, which includes the sequential stages of data collection, engagement and reach analysis, performance visualization, content design analysis, and results synthesis.

Following the data collection of 157 human-like female VPs, the study proceeded to the Engagement and Reach Analysis phase, which was divided into Image Analysis and Video Analysis components.

In the Image Analysis, engagement performance was assessed using like rate only, because Instagram does not provide observable impressions counts for image posts in the dataset. Like rate was treated as a limited proxy for passive interaction intensity rather than as a direct measure of relational bonding. Profiles were prioritized for closer review when at least 1 image exceeded a like rate of 1.0, indicating that the post generated more likes than the account’s total follower base and therefore represented an extreme interaction outcome. For these cases, the analysis compared recurring profile characteristics, most notably CGI-identifiable versus photorealistic styling, the degree of character and behavioral consistency across visual content and captions, and the frequency of copresence in the highest performing image content.

The Video Analysis retained like rate as a relative interaction metric and additionally examined high-impression distribution events using Instagram’s video impression data. Rather than relying on follower-normalized reach, the analysis concentrated on videos surpassing 20 million impressions and then profiled the accounts and posts responsible for these events. Because Instagram strategically prioritized short-form video (ie, Reels) during the study period, these reach outcomes should not be interpreted as reflecting only intrinsic content superiority; they likely also reflect platform-level distribution bias favoring Reels. Accordingly, the video analyses distinguish between interaction-based performance (like rate) and distribution-based performance (impressions).

The 20 million impression threshold was selected as a dataset-specific marker of extreme reach within the sampled population of female human-like VPs. The purpose of this threshold was to identify a subset of genuinely overperforming posts that was still sufficiently small to permit meaningful pattern-based comparison. Lower cutoffs, such as 10 million impressions, captured a substantially larger share of the sample and therefore reduced the discriminative value of the category. Accordingly, the 20 million threshold was used as a pragmatic analytic criterion to isolate rare, high-salience distribution events within this specific VP niche, rather than as a universal benchmark of Instagram virality.

To limit sensitivity to one-off anomalies (eg, public relations–driven boosts or amplification through exceptionally large nonvirtual cocreators), each account’s performance was analyzed using (1) its single best-performing image and video post, and (2) the mean of the 3 best posts per format for like rate and impressions.

Following performance identification and visualization, the content design analysis qualitatively assessed the design features of the most extreme-performing posts, with emphasis on identity coherence, CGI-likeness versus photorealistic ambiguity, human-VP copresence, and recurring patterns involving dark hair, dark eyes, and exaggerated female body types.

Finally, in the results synthesis and discussion, evidence from image engagement patterns, video engagement, and high-reach events, and the content design review was integrated to address the study’s exploratory propositions and to derive empirically grounded implications for how human-like female VPs achieve engagement and reach on Instagram.

### Ethical Considerations

Data collection was limited to publicly available profile- and postlevel information relevant to the research questions and was conducted in accordance with platform accessibility at the time of collection. One major ethical consideration was the responsible use of public information and the avoidance of unnecessary republication of identifiable account content beyond what was required for scientific analysis. Data collection was limited to information pertinent to the research objectives and was performed in strict accordance with Instagram’s terms of service and public data policies. Furthermore, all information used for this study is freely available in the public domain; did not involve human participants, their tissues, and/or data; does not involve methods or subject matter that is sensitive; and is deemed not human subjects research [27]. Moreover, visual evidence is included strictly as analytical data. Because this study evaluates complex, nonstandard visual traits, such as explicit CGI-likeness, photorealistic ambiguity, and exaggerated sexual dimorphism, including representative visual examples is methodologically necessary to operationalize these variables for the reader and ensure the reproducibility of our content design analysis.

### Participants and Procedure

The data collection process for this study was designed to identify public Instagram accounts representing human-like VPs for subsequent quantitative and qualitative analysis. For the purpose of this study, “human-like” was defined as exhibiting coherent human morphology (head, torso, and limbs) and facial features capable of recognizable human expression and gaze. This definition includes VPs with stylized aesthetics, such as anime-inspired avatars or exaggerated body proportions, provided that they retained a fundamentally human anatomical structure and human-like behavioral presentation. By contrast, abstract virtual characters—such as nonhumanoid objects, animals, or geometric entities lacking standard human biological features—were excluded.

During the initial screening stage, both human-like female VPs and human-like male VPs were identified. However, only 5 human-like male VPs met the eligibility criteria, which was too few to support meaningful subgroup comparison. To preserve analytical consistency and avoid unstable comparisons across highly imbalanced gender groups, only human-like female VPs were retained in the final dataset. Thus, the final analytic sample comprised 157 human-like female VPs, whereas the 5 human-like male VPs identified during screening were excluded from further analysis.

To reduce the likelihood of including heavily manipulated or commercially repurposed accounts with inflated audience metrics, only profiles with more than 100,000 followers were included in the final sample [28,29]. This threshold was selected because follower-purchase services have made it relatively easy to inflate lower-tier accounts, whereas manipulation of macro- and mega-level profiles is generally more detectable through content chronology gaps and third-party analytic indicators. Nevertheless, the possibility of residual manipulation cannot be excluded entirely [9,30,31].

The resulting dataset comprised 157 human-like female VPs. The restriction to macro- and mega-level accounts improved comparability within the final sample, but it also means that the study describes performance patterns among established incumbents rather than growth trajectories among emerging profiles.

Posts selected for detailed qualitative content review were identified using predefined quantitative thresholds applied to the full dataset. Specifically, posts were flagged for structured qualitative review when they met at least one of the following criteria: (1) image or video like rate greater than 1.0, indicating that the post received more likes than the account’s total follower count; or (2) video impressions greater than 20 million, indicating an extreme distribution event within the sampled VP category. In this manuscript, “structured qualitative review” refers to manual assessment of coded visual and narrative variables using the predefined coding framework, including visual realism, identity consistency, copresence structure, appearance traits, body-type representation, and production technique where relevant. The process did not involve open-ended interpretive coding beyond these predefined variables.

## Results

### Overview

To contextualize the characteristics observed among the highest performing subsets, baseline descriptive statistics were compiled for the full coded sample of 157 human-like female VPs. The distribution of all coded profile-level variables, including visual realism, identity consistency, appearance patterns, and body-type representation, is provided in Table S2 in Multimedia Appendix 1. These full-sample descriptive statistics serve as the reference context for interpreting the traits observed among the highest performing profiles and posts.

### Image Engagement Analysis (Like Rate)

**Figure 2.**
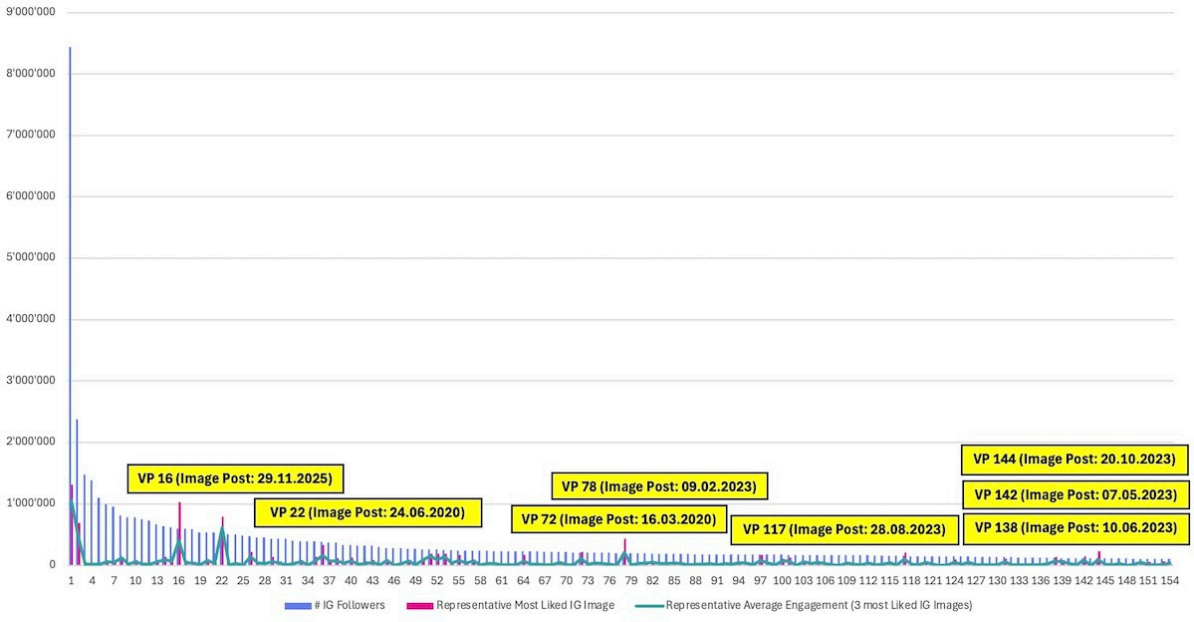
Image likes and engagement metrics. VP: virtual profile; IG: Instagram.

The image engagement analysis identified 8 top-performing human-like female VPs that produced posts with like rates greater than 1.0, indicating unusually strong interaction intensity relative to audience size (Figure 2). By contrast, several legacy CGI-like profiles with very large follower bases performed strongly in terms of absolute like volume but less strongly relative to like rate terms, because interaction levels scale less than proportionally with follower count. This distinction separates relative engagement from scale-dependent absolute interaction and provides a descriptive basis for the subsequent content review of visual and narrative features commonly observed alongside unusually high like rate outcomes.

### Image Content Design Analysis

The high like rate image posts examined in this analysis reveal several recurring visual and narrative patterns observed in posts with exceptional relative engagement (see Figure S1 in Multimedia Appendix 2 for visual evidence of these design variables). A prominent feature across the sample is copresence within the image frame: in half of the top-performing images, more than 1 subject is depicted, which represents a recurring pattern observed in posts with high relative engagement. Importantly, this pattern is not limited to the inclusion of real humans. While the informal car scene featuring the VP 16 alongside a prominent human celebrity athlete represents a particularly salient example of celebrity copresence appearing alongside higher engagement, it is not the only pattern associated with high performance.

Rather, the broader pattern suggests that multiple subjects—whether human-VP or VP-VP copresence—were frequently observed in posts with higher interaction intensity, possibly through increased perceived social density and enhancing narrative richness. In the case of CGI-like VP 72, relative engagement may also have been related to platform-level social signaling of mega VP 2 who is both visibly featured in the image post and liked the post too, which in combination was observed alongside higher relative engagement. This distinction underscores that social validation mechanisms operate both within and outside the visual frame.

Across the image set, stylistic and character consistency emerges as a common attribute, particularly among CGI-like profiles. Because such characters are typically maintained as stable 3D models or tightly controlled generative assets on creators’ servers, they allow for high visual continuity across VP content. This technical consistency may contribute to recognizability and parasocial familiarity. Topical consistency in captions was also prevalent in these posts, as illustrated by VP 22, where narrative alignment between visual content and text co-occurred with stronger audience resonance and engagement. The CGI-identifiable VP 22 also achieved high like rate without relying on any character copresence, indicating that explicit artificiality does not preclude strong performance.

From an aesthetic standpoint, most high-performing images depict VPs with dark hair and dark eyes, with 6 of the 8 highlighted profiles fitting this pattern and the remaining 2 featuring blonde characters. Compared with the baseline frequency of 62.42% in the full sample (Table S2 in Multimedia Appendix 1), this represents a descriptive concentration of dark-featured VPs among the highest performing image posts. While causal inference is not possible from this descriptive analysis alone, this concentration may reflect prevailing audience taste structures within the dominant follower demographics. In addition, many of the images emphasize exaggerated female body features, particularly pronounced bust and waist-hip contrast, as exemplified by VP 142 (Figure S1 in Multimedia Appendix 2). Such visual exaggeration, bordering on pornographic stylization, may reflect content designed for a predominantly male audience, a dynamic that is further illustrated by the VP 138 post, which situates 2 stylized human-like female VPs in a construction-site setting wearing safety vests and helmets. The juxtaposition of sexualized female forms with traditionally male-coded occupational contexts may attract attention through contrast and stereotype activation.

The image-level findings indicate that higher relative engagement was more commonly observed alongside a combination of physical and behavioral consistency, thematic coherence, and audience-targeted visual framing. Copresence, whether enacted visually or reinforced through platform-level interactions, emerges as a particularly robust pattern, suggesting that perceived social density and narrative context were frequently observed in posts with high relative engagement on Instagram.

### Viral Video Reach Analysis (Impressions)

**Figure 3.**
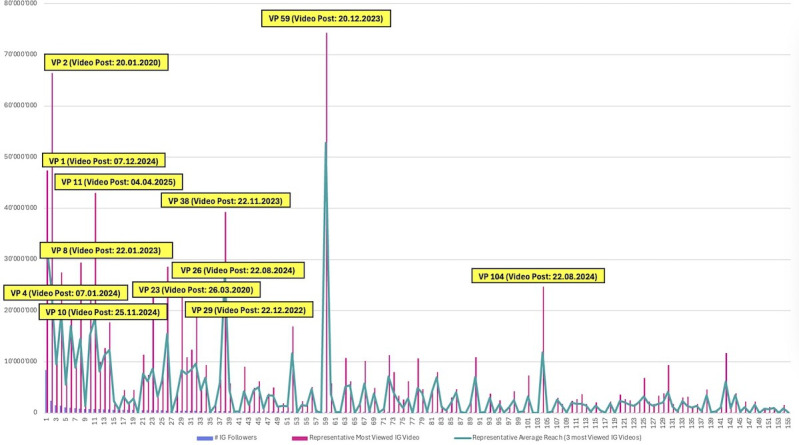
Video impressions and reach metrics. VP: virtual profile; IG: Instagram.

The analysis of viral video reach indicated a strong concentration of extreme virality within a limited subset of human-like VPs (Figure 3). Videos exceeding 20 million impressions were produced exclusively by a relatively small number of established accounts, underscoring a pronounced scale effect in which many of the most viral videos originated from profiles with the largest follower bases. At the same time, a very small number of lower-follower outliers also exceeded the 20 million impression threshold. These exceptions suggest that extreme video reach was not exclusively limited to the largest accounts, although some cases appeared likely to involve external amplification mechanisms such as a brand collaboration.

### Viral Video Content Design Analysis (Impressions)

Analysis of viral video content exceeding 20 million impressions reveals a clear descriptive pattern observed among posts with extreme reach performance (see Figure S2 in Multimedia Appendix 2 for visual evidence of these design variables). Most notably, CGI-like VPs dominate the highest performing videos, with 7 of the 12 most viral videos produced by CGI-like VPs. This qualified majority indicates that explicitly legible CGI aesthetics were commonly observed among exceptionally viral videos. This prevalence of CGI aesthetics among viral videos (58%) is notably higher than the baseline of 8.92% observed in the full sample, suggesting that explicitly virtual identities were more common among extreme reach outcomes. In contrast to photorealistic VPs, whose motion-based content may expose perceptual inconsistencies, CGI-like profiles exhibit structurally stable identity representation, which was frequently observed in video contexts where frame-to-frame coherence is critical.

As discussed in the image engagement analysis, CGI-like VPs inherently exhibit a high degree of visual and behavioral consistency, as they are typically maintained as persistent 3D assets or tightly controlled generative models. This consistency may help describe their prominence among high-performing videos by reinforcing recognizability and thus perceived authenticity and may coincide with algorithmic amplification and sustained viewer attention. The dominance of CGI-like profiles among the most viral videos suggests that, for reach-driven outcomes, clarity and stability of artificial identity were more commonly observed than incremental photorealistic gains among these high-reach videos.

Within the viral video set, there is also evidence consistent with a positive descriptive role for VP-human copresence, consistent with prior findings [32]. One particularly salient example is the video by VP 38, which achieved nearly 40 million impressions and features the VP alongside a real human. Importantly, VP 38’s appearance remains unmistakably CGI-like; rather than diminishing performance, this explicit artificiality appears alongside high audience reach. The juxtaposition of a CGI-like character with a real person may be perceived as reinforcing authenticity, transparency, and originality, aligning with prior observation that human copresence has been linked to higher engagement when the virtual nature of the VP is not ambiguous [32].

Beyond copresence, the broader visual patterns observed in viral videos largely mirror those identified in the image content design analysis. Characters with dark hair and dark eyes appeared repeatedly among the top-performing posts examined in this subset. In addition, physically feminine body types, often emphasizing large busts and wide hip-waist ratios, as well as female stereotype activating contexts, were also commonly observed in this subset. These descriptive patterns may reflect dominant audience preferences.

Ultimately, the case of VP 104 represents a notable and theoretically informative outlier. Despite a comparatively small follower base, this profile achieved exceptional video reach, positioning it alongside much larger accounts. Like the CGI-like profiles, VP 104 is unambiguously virtual, but her morbidly obese body type departs sharply from conventional influencer aesthetics. This deviation may have co-occurred with high impression counts through a combination of radical transparency, visual novelty, and perceived authenticity. It may also reflect spectacle- or ridicule-based entertainment dynamics associated with exaggerated motion and bodily contrast. Regardless of the underlying mechanism, this case is consistent with a central observation: in contemporary Instagram video environments, explicit virtuality, rather than photorealistic ambiguity, was frequently observed as a characteristic of extreme video reach.

### Video Engagement Analysis (Like Rate)

**Figure 4.**
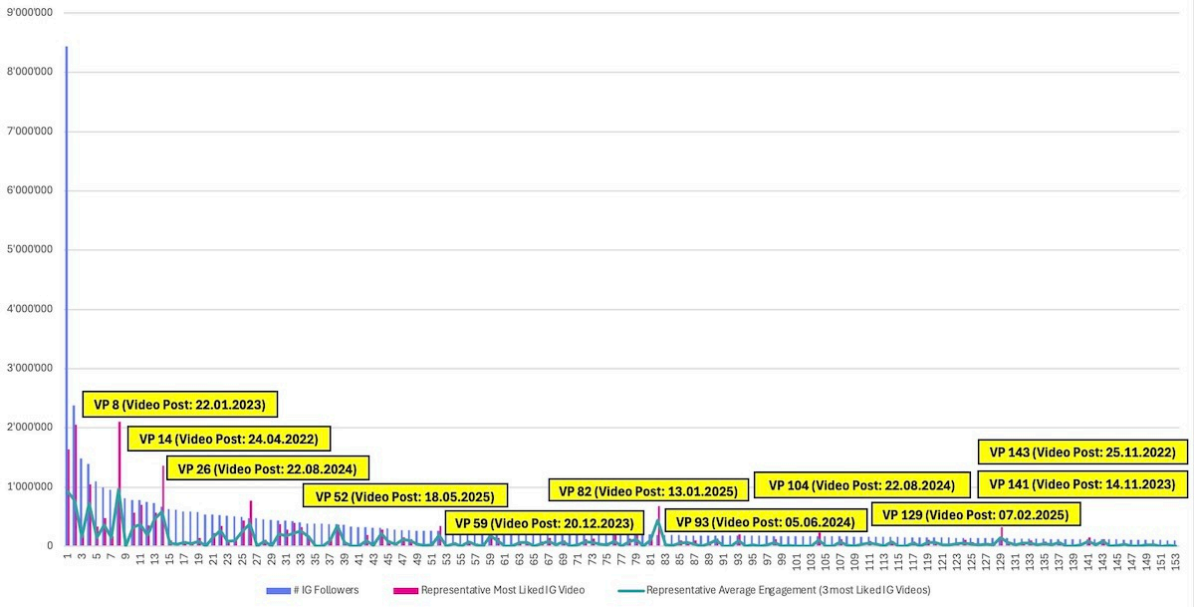
Video likes and engagement metrics. VP: virtual profile; IG: Instagram.

The video engagement analysis, which was based on relative like rate rather than absolute reach, identified a partially overlapping but clearly distinct group of high-performing VPs (Figure 4). Compared with the viral video reach analysis, only a subset of accounts appeared in both categories, indicating that distribution-based success and interaction-based success followed different descriptive patterns within the sample. This divergence is consistent with the distinction between reach and engagement: absolute impressions heavily represent profiles with large existing audiences and algorithmic momentum, whereas like rate normalizes interaction by follower count and therefore reduces the scale-dependent advantage.

### Video Content Design Analysis (Like Rate)

In contrast to the viral video reach analysis, the video engagement analysis reveals a markedly different pattern with regard to top-performing content design (see Figure S3 in Multimedia Appendix 2 for visual evidence of these production techniques). Only a small subset of CGI-like VPs, VP 59 and VP 82, appeared among the posts with the highest interaction intensity, indicating that CGI-like VPs were more commonly observed in high-reach distribution events than in high-engagement interactions. This divergence indicates that CGI-like profiles were commonly observed in large-scale exposure events, whereas their ability to convert visibility into active engagement is more limited.

Instead, the video engagement results are dominated by photorealistic VPs, including VP 8, VP 14, VP 26, VP 52, VP 93, VP 104, VP 129, VP 141, and VP 143. While photorealistic profiles constituted 91.08% (143/157) of the total sample, they represented only a minority (5 out of 12) of top-performing videos by reach rate but a majority (9 out of 11) of top-performing videos by like rate, indicating a descriptive shift toward realism for interaction-based success. These profiles are more commonly observed among the top-performing videos in terms of like rate and predominantly rely on generative AI video techniques or face-swapping software, such as the HelloFace app, to map highly realistic virtual faces onto human bodies. This approach may be perceived as increasing realism, emotional expressiveness, and micro-level social cues, and these features were commonly observed in videos with high active user engagement. Notably, this finding represents the inverse of the reach-based results, where CGI-like profiles were dominant, underscoring that reach and engagement correspond to distinct design mechanisms.

An additional and noteworthy pattern concerns the strong engagement performance of VP 52, VP 104, and VP 141, all of which embody morbidly obese virtual personas. These profiles were observed among posts with exceptional like rates despite diverging sharply from conventional virtual beauty standards. The reasons underlying this outcome remain ambiguous and may involve perceived authenticity, parody, or even elements of ridicule-driven entertainment; however, the data do not allow for a definitive attribution.

Across these high-performing videos, several shared visual and behavioral characteristics emerge. The content typically zooms in on the VP’s face and upper body, allowing for clear display of facial expressions, eye contact, and subtle emotional cues. These nonverbal behaviors are known to facilitate perceived social proximity, parasocial bonding, and active response [33], which aligns with our observation of these features in high-engagement videos. Consistent with both image engagement and viral video reach analyses, the high-performing video engagement content largely features VPs with dark hair and dark eyes. The primary exceptions are the blonde profile of VP 26, the stylized Asian avatar with pink-dyed hair and bright contact lenses of VP 82, and the light-eyed yet dark-haired profile of VP 14. These results reinforce the notion that certain visual traits recur across performance dimensions.

## Discussion

### Principal Findings

#### Overview

This study evaluated exploratory propositions concerning reach and engagement among human-like VPs on Instagram and identified several recurring descriptive patterns across image and video content. Overall, the findings indicate that identity and behavioral consistency were commonly observed among high-performing content across formats. Photorealism alone did not emerge as a uniform marker of stronger performance; rather, the observed patterns aligned closely with transparent virtual presentation, identity coherence, and format-specific content strategies. Human-VP and VP-VP copresence both appeared among high-performing posts, although their role varied by format and should be interpreted descriptively. Moreover, the study findings should be interpreted as descriptive associations within the sampled dataset rather than as statistically confirmed differences or causal effects.

#### Image Content Engagement Evaluation

The image engagement analysis indicates that high like rate was commonly observed alongside copresence within the image frame, strong identity and narrative consistency, and audience-targeted visual framing. CGI-like profiles also appeared among high-performing images, including in cases without copresence. Across profiles, dark hair and eyes and exaggerated feminine stylization recurred repeatedly, indicating a descriptive concentration of these traits among high-performing image posts.

From a theoretical perspective, the findings align with social identity theory, whereby contextual, physical, and behavioral congruence between VPs and their audiences fosters identification and self-affirmation. Prior research on parasocial relationships and digital intimacy suggests that sustained, 1-sided engagement with idealized figures may contribute to distorted relationship expectations, emotional overidentification, and social comparison effects [33,34]. The frequent emphasis on exaggerated body features may further intensify appearance pressures.

Identity coherence emerges as a foundational characteristic, particularly for CGI-like VPs. The use of stable 3D models or tightly controlled generative assets supports high visual and behavioral consistency, which may contribute to recognizability and parasocial familiarity over time. When captions aligned topically with imagery, stronger audience resonance was descriptively observed, indicating that narrative continuity across modalities was observed alongside stronger audience resonance. Importantly, CGI-like profiles that retain sufficient human resemblance to avoid “uncanny valley” discomfort were observed among high-performing posts [35], suggesting that stronger engagement was more commonly observed with a balance of authenticity and transparency rather than maximal photorealism alone.

Copresence operates both directly (image post human-VP or VP-VP cofeaturing) and indirectly through platform-level social signaling activity. These patterns show that higher relative image engagement was commonly observed alongside social-contextual cues that reflect high perceived social density and narrative consistency. Copresence increases interpretive depth and invites social inference, which may coincide with interaction. Platform-level validation (eg, likes from prominent accounts) further reinforces this pattern by signaling relevance and credibility beyond the visual content itself.

Overall, image engagement was more commonly observed alongside the interplay of consistent character and contextual consistency, copresence, and audience-aligned aesthetics. These insights clarify how relative engagement manifests on Instagram while underscoring the ethical importance of transparency and responsible VP design to mitigate potential risks to digital well-being.

#### Viral Video Content Reach Evaluation

The reach-based analysis of viral video content indicates a pronounced concentration of extreme absolute reach outcomes among CGI-like VPs. Seven of the 12 most viral videos stem from CGI-like VPs, suggesting that clear CGI aesthetics and the associated stability of artificial identity were commonly observed in this subset among the high-performing viral videos. Furthermore, across the viral videos, recurring appearance cues, including dark hair and dark eyes, and highly feminized body presentations are also frequently observed, broadly aligning with the visual regularities identified in the high-engagement image content analysis.

The dominance of CGI-like VPs in viral video reach outcomes is theoretically consistent with the idea that clarity of artificial identity was frequently observed in video contexts. Motion content increases perceptual demand: viewers continuously evaluate facial dynamics, eye behavior, and body physics, meaning that near-photorealistic VPs may be penalized if small inconsistencies accumulate across frames. CGI-like VPs, by contrast, implicitly set a different expectation baseline and often exhibit higher frame-to-frame coherence because they are maintained as persistent 3D assets or tightly controlled digital low-rank adaptation models. This structural consistency may contribute to recognizability and lower perceptual friction, which in turn may coincide with greater watch time and reexposure, signals that frequently co-occur with algorithmic amplification and were frequently observed among viral video content on Instagram.

Finally, within this set, very high reach was also observed in selective cases featuring VP-human copresence, with VP 38 reaching nearly 40 million impressions in a hybrid scene, consistent with the observation by Cascio et al [32] that human copresence can strengthen VP content performance when virtuality remains unambiguous. However, the present findings suggest an important boundary condition: copresence appears most effective when it reinforces rather than obscures the VP’s synthetic ontology. Meaning, hybrid compositions may be especially potent when the contrast between human and virtual increases perceived transparency, authenticity, and narrative tension, and may co-occur with stronger audience attention and algorithmic distribution. In summary, the viral video content reach analysis suggests that extreme video virality among human-like VPs was commonly observed alongside explicit virtual legibility, identity stability, and high-salience design cues, and that the “uncanny valley” concept may contribute to reach both by being avoided (through clear CGI stylization) and, in some cases, by being exploited as a mechanism of discomfort-driven reach.

#### Video Content Engagement Evaluation

Video content overall showed higher interaction intensity than static images, with 11 video posts exceeding a like rate of 1.0 compared with 8 image posts, underscoring the heightened persuasive power of motion-based content [33,34]. Contrary to the viral video reach analysis, the video engagement analysis reveals a distinct performance structure. Unlike viral video reach, high video engagement is not primarily driven by CGI-like profiles. Only a small subset of CGI-like VPs, VP 59 and VP 82, appears among the top performers in terms of like rate. Instead, the majority of high-engagement videos are produced by photorealistic VPs using advanced generative AI or face-swapping technologies, indicating that engagement and reach were characterized by different design patterns.

The divergence between reach and engagement outcomes suggests that algorithmic exposure and active user endorsement may reflect different perceptual pathways and platform dynamics. CGI-like profiles, which dominate extreme reach events, appear less commonly observed in converting visibility into proportional engagement. This pattern suggests that their virality may coincide with attentional capture, potentially through novelty, salience, or discomfort, rather than affective affinity. In line with the viral video reach evaluation, this is compatible with the post hoc interpretation that CGI-like virality may, in some cases, reflect attention capture associated with perceptual incongruity, whereby perceptual incongruity sustains attention and impressions without consistently translating into positive endorsement [35].

By contrast, video content engagement is clearly dominated by photorealistic VPs. Seven of the 11 top-performing videos with regard to like rate exhibit full photorealism consistent with face-swapping and advanced generative AI technologies, suggesting that such techniques were more commonly observed than more complex CGI tools among posts with high interaction. The relative visual homogeneity of these videos suggests that audiences may respond particularly strongly to realistic motion, facial expressiveness, and microemotional cues, which are known to facilitate parasocial bonding and active response [33].

A theoretically informative exception is VP 104, which performs exceptionally well in terms of both reach and engagement, unlike most CGI-like VPs that display a clear asymmetry between these metrics. Although photorealistic, the VP is unmistakably artificial and departs sharply from conventional influencer aesthetics through a morbidly obese body type. This combination of clear virtuality and extreme body type appears to mitigate the negative effects typically associated with the “uncanny valley,” suggesting that perceptual discomfort does not necessarily suppress either attention or interaction in this case. Instead, VP 104—together with the thematically aligned profiles of VP 52 and VP 141—may benefit from radical transparency, novelty, and emotional arousal, co-occurring with both high visibility and active user response.

Across high-engagement videos, recurring design features further clarify these dynamics. Content typically centers on the VP’s face and upper body, enabling direct gaze, visible facial expressions, and subtle emotional signaling, nonverbal cues commonly discussed in relation to perceived social proximity and parasocial interaction [36]. Notably, only one of the high-engagement videos features VP-VP copresence; instead, interaction intensity was often observed in solo-performer formats, where audience attention may be concentrated on a single virtual persona. This contrasts with the static image-based findings, where copresence played a more prominent role, and further underscores that image and video are characterized by different content design patterns. Consistent with image engagement and video reach analyses, VPs with dark hair and dark eyes appeared frequently in the high-performing subset, with limited stylistic exceptions (eg, the blonde VP 26, pink-dyed VP 82, and the bright-eyed VP 14), indicating that certain visual traits retain cross-format relevance.

Taken together, these findings indicate that video is the most effective medium for generating engagement, but that success depends critically on how virtuality is expressed. CGI-like content appears optimized for reach—potentially through reverse “uncanny valley” dynamics—whereas photorealistic, face-swapped videos are more commonly observed in content that paired attention with interaction. The case of VP 104 demonstrates that when artificiality is both explicit and narratively coherent, the “uncanny valley” need not constrain performance, highlighting important boundary conditions for theories of virtual embodiment and audience response.

#### Additional Findings

Beyond the formal research propositions, the study yielded several additional findings that further illuminate how human-like VPs generate engagement and reach across image and video content formats. A central observation is that image and video performance feature fundamentally different VP types and design cues, indicating that success was not observed uniformly across media. The characteristics commonly observed in high-performing video content differed from those commonly observed in high-performing image content, reinforcing the importance of distinguishing algorithmic exposure from active user response.

Although no single configuration of physical or ethnic attributes emerged as a universal descriptive marker of success, a clear concentration of VPs with dark hair and dark eyes was observed among top-performing profiles across both image and video formats. We hypothesize that this pattern may correspond to the physical traits of major segments of the global population, particularly within Asian, African, Middle Eastern, and Latin American regions, which together constitute the numerical majority of social media users [34]. From a social identity perspective, audiences might therefore be more likely to engage with VPs that visually resemble their own or reflect familiar, normative appearance cues [18]. However, as this study did not use specific audience demographic data, we cannot confirm that this design trend results from audience self-identification. Consequently, this interpretation remains a theoretical possibility for future research rather than a conclusion supported by the current data.

Importantly, the findings indicate that there is no homogeneous blueprint for a universally high-performing human-like VP. While certain trends recur, the remaining design attributes among top-performing profiles are highly heterogeneous. This diversity suggests that engagement is contingent on contextual coherence and audience fit, rather than strict adherence to a fixed aesthetic formula. Consistent with this, no single VP appeared among both the top-performing image and video posts, pointing toward content format specialization rather than cross-format dominance.

Another noteworthy outcome is that some highly viral video posts were produced by VPs with average or below-average overall following, including VP 59 and VP 104. This challenges the misconception that VPs with only above-average-follower counts can generate viral content. Instead, it suggests that content-specific relevance, emotional salience, and algorithmic amplification may coincide with episodic virality beyond baseline profile performance.

From a digital health and behavioral science perspective, these dynamics highlight the emergence of nonlinear influence patterns in AI-mediated environments. The fact that below-average-follower VPs can intermittently generate extreme reach or engagement underscores how affective design cues and platform algorithms jointly shape exposure and psychological outcomes. Such mechanisms raise concerns related to the attention economy, emotional contagion, and the amplification of content that may provoke strong reactions without delivering informational or social value—dynamics previously linked to parasocial overidentification and mental well-being risks [36].

The linguistic analysis further revealed a strong dominance of English across all content formats, which may reflect algorithmic prioritization rather than pure audience preference. Whether this linguistic dominance aligns with user needs, particularly in health communication contexts, remains an open question.

Finally, the observation that top-performing videos were generally created more recently than top-performing images highlights the rapid evolution of generative video technologies and the historical lag that previously constrained AI video production. As these tools continue to mature, their persuasive capacity, and associated risks, will expand accordingly. Prior research has shown that realistic motion and expressive facial behavior can significantly enhance engagement [33], but such psychological potency also introduces ethical challenges related to emotional manipulation, misinformation susceptibility, and addictive engagement loops [37,38].

Taken together, these additional findings reinforce the need for cross-sector collaboration between technologists, health communication researchers, and policymakers. As human-like VPs become increasingly lifelike and socially influential, future research must move beyond surface-level engagement metrics to assess psychological, social, and ethical implications. Improving transparency in content provenance, strengthening verification protocols, and establishing responsible design standards will be critical to ensuring that AI-generated personas support digital engagement without undermining user well-being, trust, or public health outcomes.

#### Implications of Findings

This study offers substantial insights not only to traditional modeling agencies and human influencers but also to broader fields concerned with digital communication, health promotion, and the ethical design of AI-generated personas. The data underscore the importance of transparent, authentic, and narratively consistent content in relation to engagement outcomes, suggesting that creating coherent and identifiably artificial human-like VPs may help inform how commercial and public organizations navigate the ongoing digital transformation. In health-related contexts, similar approaches could be used to design perceived trustworthy and authentic virtual health educators or support agents that may capture attention and sustain engagement while maintaining transparency about their artificial nature.

Using VPs as a testing ground for audience response also offers opportunities beyond marketing. Agencies, and by extension, health communication practitioners, can identify which visual, behavioral, and linguistic attributes elicit trust, empathy, or attention, providing valuable insights for developing more effective virtual counselors, digital therapy companions, and patient education interfaces. This mirrors evidence from human-computer interaction studies showing that perceived authenticity and emotional resonance strongly influence both adherence and behavior change [39].

For digital creators and VI agencies, the findings highlight that VPs combining consistent physical, behavioral, and narrative coherence are commonly observed among profiles showing sustained engagement. This can guide creators in refining design parameters that may be related to relatability, cultural alignment, and emotional comfort, all of which are equally relevant to the creation of AI health agents intended for diverse populations. The results also emphasize the potential of locally oriented VPs, those embedded within a specific cultural or linguistic context, to generate stronger audience trust through cultural congruity [40,41]. Virtual modeling and influencing agencies can leverage these principles to improve both marketability and ethical integrity, ensuring that virtual entities reflect diverse, inclusive, and psychologically safe representations.

Finally, the superior engagement performance of video content, especially when generated through face-swapping technology, suggests that motion and emotional expressiveness were frequently observed alongside high user attention. While this presents clear opportunities for content creators and educators, it also introduces psychological and ethical considerations. The same features that enhance engagement may heighten emotional persuasion or dependency. Consequently, developers of AI-driven media, including virtual health communicators, should integrate ethical safeguards, such as clear disclosure of synthetic identity, limits on emotional manipulation, and design standards that promote digital well-being.

Overall, these findings underscore the growing overlap between commercial and health communication ecosystems in an era of synthetic media. As virtual personas become more pervasive, interdisciplinary collaboration among designers, health professionals, and policymakers will be critical to ensure that the technologies driving engagement are harnessed responsibly to educate, empower, and protect users, rather than exploit their psychological vulnerabilities.

### Comparison With Prior Work

The findings of this study broadly align with prior work on VIs and human-AI interactions while refining several points of interpretation. Rather than identifying visual realism itself as the primary feature of interest, the present results suggest that transparent virtual presentation, consistency of identity, and format-specific expressive cues were closely aligned with the observed higher performance. These conclusions remain descriptive and should be interpreted in relation to the present sample of established human-like female VPs.

Extant studies have reported that increasing human-likeness in VIs can, under certain conditions, reduce positive reactions and trigger uncanny valley effects. In contrast to interpretations that emphasize realism itself as problematic, our results show that ambiguity in virtual identity was the primary factor linked to apparent perceptual discomfort. Photorealistic VPs in this study did not exhibit reduced engagement when their artificial nature was clearly communicated, suggesting that uncanny valley effects are context-dependent and contingent on transparency rather than visual fidelity alone [5,11,35].

This finding suggests that concerns related to the uncanny valley may be highly context-sensitive: in social and influencer environments—where users anticipate artificial mediation—explicit virtual legibility can mitigate perceptual discomfort and frequently co-occurs with higher engagement [6,20]. From a health communication perspective, this insight has implications for designing trustworthy virtual health agents or AI health educators, where credibility is supported less by realism itself than by ethical framing, transparency, and behavioral consistency [14,15,27].

Authenticity and consistency also emerged as central factors prevalent among high-engagement content. Prior studies have identified authenticity as a core factor associated with VI success [7,8,19]. Our data extend this understanding by demonstrating that coherence across physical, ethnic, and behavioral cues was frequently observed among posts with stronger engagement outcomes [10,12]. This pattern aligns with social identity theory [18], which posits that individuals are more likely to connect with figures who reflect aspects of their own identity. The resemblance between high-performing VPs and the ethnic characteristics of their primary audiences exemplifies this process and translates it into measurable behavioral responses [16,20]. These results parallel findings in digital health research showing that cultural and linguistic alignment strengthens trust and adherence in AI-mediated patient interactions, underscoring the cross-domain relevance of identity congruence [24,25,39].

Our findings partially diverge, rather than fully contradict, prior expectations regarding human-virtual copresence. While earlier work suggested that human inclusion may enhance credibility and social realism [12,16], this study shows that human-VP copresence is not inherently superior to VP-VP copresence, and that both frequently co-occurred with strong engagement and reach when the VP’s artificial nature remains clearly identifiable [20,32]. Notably, the most engaging image posts did not rely on human collaborators, suggesting that audiences may value virtual autonomy and narrative coherence over hybrid realism. In digital health contexts, this insight can inform the design of autonomous virtual counselors or therapeutic avatars that cultivate trust through identity stability and emotional presence rather than simulated human companionship [15,23,27].

Finally, the results reinforce earlier evidence of the growing dominance of video content [33]. Engagement with video posts far exceeded that of image posts, particularly when videos were generated using AI-driven face-swapping tools that produce emotionally legible motion rather than maximal photorealism [4,6,32]. This distinction aligns with the study’s broader finding that expressive motion, microemotional cues, and behavioral realism were more consistently observed among top-performing content than visual realism alone. While this trend holds promise for enhancing health communication reach and interactivity, it also underscores the need for ethical guidelines governing the persuasive potential of emotionally powerful AI media—especially in domains where user vulnerability, misinformation, or emotional influence may carry public health implications [9,27,37].

### Limitations

A major limitation of this research lies in the potential algorithmic bias introduced by the usage of the authors’ personal Instagram account during the data generation phase. Although significant efforts were made to ensure the consideration of a diverse range of profiles during data collection, it must be acknowledged that Instagram’s algorithm may have skewed the selection process by potentially prioritizing profiles that relate to the authors’ previous browsing activity. To mitigate this limitation in future research, it is advised to create novel unbiased Instagram accounts used for the purpose of research only. This would enable the collection of a more potent and representative dataset.

A further limitation concerns the operationalization of engagement solely through like rate. Likes reflect low-effort, passive approval and do not capture more active forms of relational response such as commenting, sharing, or sustained conversational interaction. In addition, like rate normalization may disadvantage legacy accounts with large numbers and inactive followers relative to newer accounts whose follower base is more active. Accordingly, the engagement findings should be interpreted as patterns in passive interaction intensity rather than as comprehensive measures of audience bonding or trust.

Furthermore, the exclusion of profiles with fewer than 100,000 followers introduces a survivor bias. By limiting the analysis to macro- and megainfluencers to avoid manipulated accounts, the study effectively examines the characteristics of established success rather than the factors contributing to growth. Traits observed among high-performing macro- and mega-level accounts may not generalize to early-stage profile development.

Additionally, the comparative analysis of content formats must account for platform-level distribution biases. During the data collection period, Instagram’s algorithm heavily prioritized short-form video (ie, Reels) to compete with emerging market competitors, resulting in systemic amplification of video content over static images. Consequently, the superior reach and engagement observed for video posts may partially reflect this strategic platform prioritization rather than purely intrinsic superiority in content design or user preference. In addition, the comparison between image and video performance must be interpreted in light of platform-level distribution asymmetry. During the data collection period, Instagram’s prioritization of Reels likely increased the exposure potential of video content independently of content design. The observed superiority of video on reach metrics therefore reflects both platform incentives and content characteristics.

An inherent challenge in studying VPs is their vulnerability to hacking, manipulation, or platform removal due to alleged content policy violations. This issue was exemplified by the cases of VP 5 and VP 6, whose accounts were deactivated and/or deleted during the research analysis phase. This uncertainty complicates the reliable scientific study of VPs, as there is a permanent risk of midstudy profile deletion. Fortunately, the study had included multiple alternative VPs, thus mitigating potential cluster risk, and so the analysis could be continued with immediate effect.

Moreover, the study does not directly measure audience perception of artificiality or realism in AI-generated content. As a result, it cannot empirically determine the extent to which users correctly identify photorealistic AI-generated images versus videos as synthetic, nor how such perceptions influence reach and engagement outcomes. This limitation is particularly relevant for interpreting the divergence observed between viral video reach and engagement, as well as the strong reach performance of CGI-like profiles. Without direct insight into audience-level perception, it remains unclear to what extent “uncanny valley” effects, perceptual discomfort, or ambiguity-driven attention contribute to the observed performance discrepancies.

Consequently, we offer a post hoc interpretation regarding the application of the “uncanny valley” theory [35]. While this construct typically predicts negative affect and user withdrawal, our data suggest a potential divergence between attention and affinity: the visual dissonance associated with perceived artificiality may concurrently trigger an orienting response driven by curiosity. This mechanism could elucidate why CGI-like profiles achieved high algorithmic visibility (reach) despite lower relative emotional resonance (engagement). We acknowledge this as a retrospective observation; the “uncanny valley” effect may be bidirectional in this context, where perceptual discomfort is associated with attentional capture rather than strict avoidance.

This interpretation also provides context for outliers such as VP 104. Although not CGI-like in the classical sense, the profile represents a significant deviation from conventional influencer aesthetics. Such content may achieve high reach through radical novelty or spectacle-based consumption, where viewership is motivated by expectancy violations. This suggests that for extreme reach outcomes, platform algorithms may preferentially amplify content that elicits high-arousal reactions, including potential aversion, due to their relation to retention and repeated exposure. Future research is required to experimentally isolate “attention-driven discomfort” as a distinct variable from positive narrative interest.

Finally, the speed of technological progress, combined with the fast-paced nature of the digital advertising and marketing landscape, presents a challenge for the longevity and relevance of this study’s findings. As the industry and technology advance, there is a risk that these findings may become outdated more quickly than those in other areas of management research. However, it is worth emphasizing that many of the insights presented in this study are grounded in well-established social and organizational theories, which may help sustain their relevance even as the surrounding industry continues to evolve.

### Future Research

Future research should explicitly examine user recognition, affective responses, and interpretive judgments of AI-generated content across static and motion formats. Experimental or survey-based methods are particularly well suited to clarify whether attention toward virtual content is driven primarily by attraction, repulsion, or misattribution, especially in cases where reach and engagement diverge. Such work would substantially strengthen causal inference and improve understanding of how virtual content design shapes both algorithmic amplification and human response. This line of inquiry is especially relevant given this study’s findings on CGI-like virality, where extreme reach may be associated with discomfort or novelty-based attention, rather than positive affect.

Conducting in-depth qualitative research, including audience surveys and interviews, could validate and enrich the findings of this study. Qualitative data would provide a more nuanced understanding of why certain physical attributes (such as dark hair and brown eyes within VPs) closely resonate with majority audiences, and how these aesthetic and cultural preferences might evolve over time. Rather than implying a deterministic advantage, such work could help disentangle whether these traits reflect audience self-identification, algorithmic reinforcement, or generative AI training biases. In addition to advancing marketing knowledge, such research could shed light on how exposure to idealized virtual appearances shapes user attitudes, well-being, and perceptions of authenticity.

Another area for improvement involves expanding the analysis to a broader spectrum of VP sizes, particularly micro and nano profiles. Examining smaller-scale VPs could yield valuable insights into how engagement rates vary across audience scales and the extent to which intimacy, familiarity, or social presence differs among less-followed profiles. Additionally, the creation and experimental testing of self-generated VPs on Instagram would allow researchers to systematically manipulate profile variables (such as explicit AI disclosure, identity consistency, degree of CGI stylization, or communication tone) and observe their direct effects on engagement, affect, and audience behavior.

The application of “uncanny valley” theory [35] in the context of AI-generated content remains a particularly promising line of inquiry. Building on this study’s findings, future research should not only examine avoidance-based “uncanny valley” effects but also investigate conditions under which perceptual discomfort or “wrongness” produces increased attention and reach. Experimental designs exposing participants to AI-generated images and videos with varying degrees of realism and explicit artificiality could clarify whether discomfort suppresses engagement, amplifies reach, or operates differently across content formats. Such insights would be especially valuable for the design of transparent, authentic digital health agents that balance ethical compliance with psychological comfort.

An emerging area of relevance concerns the potential for VPs to establish and promote their own brands, mirroring prominent human influencers. Future studies could examine whether VP-led brands benefit from the same trust and identity-consistency mechanisms identified in this research, or whether commercialized virtual identities introduce additional ethical and authenticity challenges. Understanding this dynamic could offer both commercial and health-focused organizations opportunities for innovation in digital identity, persuasion, and trust formation [42].

As the VI space continues to evolve, further attention should be paid to emerging human-like subcategories, particularly human-like male VPs [43], who remain highly underrepresented in the current landscape. Moreover, a particularly underexplored but empirically relevant direction concerns alternative and nonnormative virtual body types. This study identifies VP 104 and VP 141 as rare cases of VPs embodying morbidly obese physiques that nevertheless achieve exceptional video engagement and reach.

These VPs demonstrate that deviation from dominant beauty norms does not inherently suppress performance and may instead activate engagement through novelty, transparency, or emotional arousal. Future research should systematically investigate such alternative body representations, extending analysis to additional minority VPs, such as those representing individuals with Down syndrome and thereby challenging conventional beauty standards in different ways. These investigations would advance understanding of authenticity, diversity, stigma, body image, user engagement with virtual content, user anxiety, and inclusivity within AI-generated identity spaces and their potential implications for psychological well-being [26,44,45].

A significant observation from this study is the underperformance of VPs representing ethnic minorities. Currently, only a small number of macro-level Black VPs, including VP 58, VP 94, VP 100, and VP 151, achieve substantial visibility. This raisesimportant questions about the sources of this underrepresentation—whether it reflects algorithmic bias in generative AI systems or deeper societal inequalities that are being reproduced in virtual spaces. Future research should examine these factors systematically to determine whether AI adjustments and data diversification can mitigate bias, or whether more structural, societal interventions are required to promote equity and representation in the evolving ecosystem of virtual personas.

### Summary

Findings from the empirical analysis indicate that VP engagement varied notably across content formats and also co-occurred with physical and behavioral consistency, transparency, authenticity, and disclosure of artificiality. Among these factors, AI-generated video posts showed higher audience interaction and reach than static images in this dataset. These results highlight the importance of both technological sophistication and ethical compliance in shaping user response to AI-mediated social content.

### Conclusions

This study contributes to the growing literature on VIs and AI-mediated communication by examining reach and engagement patterns among established human-like female VPs on Instagram. Across sampled accounts, high performance aligned closely with identity coherence, transparent virtual presentation, and format-specific content strategies, rather than with photorealism alone.

The results further suggest that image engagement, video engagement, and video reach should not be treated as interchangeable outcomes. CGI-like presentation was frequently observed among the most widely distributed videos, whereas photorealistic and face-swapped motion content was more common among highly engaging videos by like rate. Copresence and recurring appearance stylizations also appeared repeatedly among high-performing posts, although these patterns should be interpreted as descriptive features of established accounts rather than as universal success factors.

Because the sample was restricted to macro- and mega-level human-like female VPs, the findings characterize successful incumbents and do not establish causal predictors of audience growth. Further work should extend analysis to smaller profiles, incorporate richer engagement measures, directly assess user perception of artificiality, and further examine the ethical implications of synthetic identity design in digitally mediated environments.

Finally, this study highlights the need for responsible innovation in the rapidly expanding domain of VPs. As AI-generated identities grow more pervasive and persuasive, interdisciplinary collaboration between technologists, behavioral scientists, and health communication experts will be essential to ensure that these systems are deployed not only to maximize engagement but also to safeguard user well-being, promote digital literacy, and foster trust in AI-mediated human interaction.

## Supplementary material

10.2196/86233 Multimedia Appendix 1 Content coding framework and full-sample descriptive statistics.

10.2196/86233 Multimedia Appendix 2 Visual evidence of content design variables.

## Abbreviations

AI: artificial intelligence
CGI: computer-generated imagery (displaying legible artificiality)
VI: virtual influencer
VP: virtual profile
