# Supplementary material for: Characteristics of Human-Like Virtual Profiles in Relation to Audience Reach and Engagement on Instagram: Secondary Data Analysis
Source: J Med Internet Res. 2026 Jun 5;28:e86233. doi: 10.2196/86233 (PMC13240982; doi:10.2196/86233)
Supplement: Multimedia Appendix 1 [file jmir-v28-e86233-s001.docx]

**Multimedia Appendix 1.** Content coding framework and full-sample descriptive statistics.

**Table S1.** Content design codebook (coding variables and operational definitions).

| **Variable** | **Type** | **Categories / Codes** | **Operational definition & decision rules** |
| --- | --- | --- | --- |
| **Visual Realism** | Binary | 1 = CGI-like  2 = Photorealistic | **CGI-like:** Artificial nature is visually explicit through stylized textures, non-photographic rendering, exaggerated proportions, or clearly synthetic facial features.  **Photorealistic:** Visual cues closely resemble human photographic realism and could plausibly be mistaken for real human imagery, particularly in static context. |
| **Identity Consistency** | Binary | 1 = Consistent  2 = Inconsistent | **Consistent:** Stability of facial identity, body representation, overall visual appearance, and behavioral/narrative presentation across posts. Minor variations attributable strictly to pose, clothing, lighting, or camera angle are permitted.  **Inconsistent:** Repeated and noticeable shifts in the aforementioned features that materially alter the apparent identity of the VP. |
| **Co-Presence Structure** | Categorical | 1 = Solo VP  2 = VP-VP  3 = VP-Human  4 = Not codable | **Solo VP:** One focal VP appears without any additional clearly identifiable actor.  **VP-VP:** Two or more VPs appear together within the same frame.  **VP-Human:** A VP appears alongside a clearly identifiable human individual. *(Note: Hybrid face-swapped videos showing only one apparent actor are coded as Solo VP; face-swapped videos showing a VP alongside another identifiable human are coded as VP-Human).*  **Not codable:** Visual material is insufficient to classify additional actors with confidence. |
| **Appearance Patterns** | Categorical | 1 = Dark hair and dark eyes  2 = Dark hair and bright eyes  3 = Bright hair and dark eyes  4 = Bright hair and bright eyes | Coded based on the dominant, recurring hair and eye color combinations of the VP. Treated as descriptive visual traits capturing stylization trends, not fixed biological classifications. |
| **Body Type Representation** | Binary | 1 = Exaggerated  2 = Non-exaggerated | **Exaggerated:** Visually observable exaggerated sexual dimorphism, specifically markedly amplified bust-to-waist and/or hip-to-waist contrast relative to surrounding body proportions depicted in the account’s content.  **Non-exaggerated:** Absence of extreme visual amplifications. |

**Table S2.** Full-sample descriptive characteristics of the 157 coded human-like female virtual profiles.

| **Variable** | **Category** | **n** | **%** |
| --- | --- | --- | --- |
| **Visual Realism** | CGI-like | 8.92 | % |
|  | Photorealistic | 91.08 | % |
| **Identity Consistency** | Consistent | 77.71 | % |
|  | Inconsistent | 22.29 | % |
| **Appearance Patterns** | Dark hair and dark eyes | 62.42 | % |
|  | Dark hair and bright eyes | 5.10 | % |
|  | Bright hair and dark eyes | 15.92 | % |
|  | Bright hair and bright eyes | 16.56 | % |
| **Body Type Representation** | Exaggerated | 55.41 | % |
|  | Non-exaggerated | 44.59 | % |
