# Supplementary figures and images for: Characteristics of Human-Like Virtual Profiles in Relation to Audience Reach and Engagement on Instagram: Secondary Data Analysis
Source: J Med Internet Res. 2026 Jun 5;28:e86233. doi: 10.2196/86233 (PMC13240982; doi:10.2196/86233)

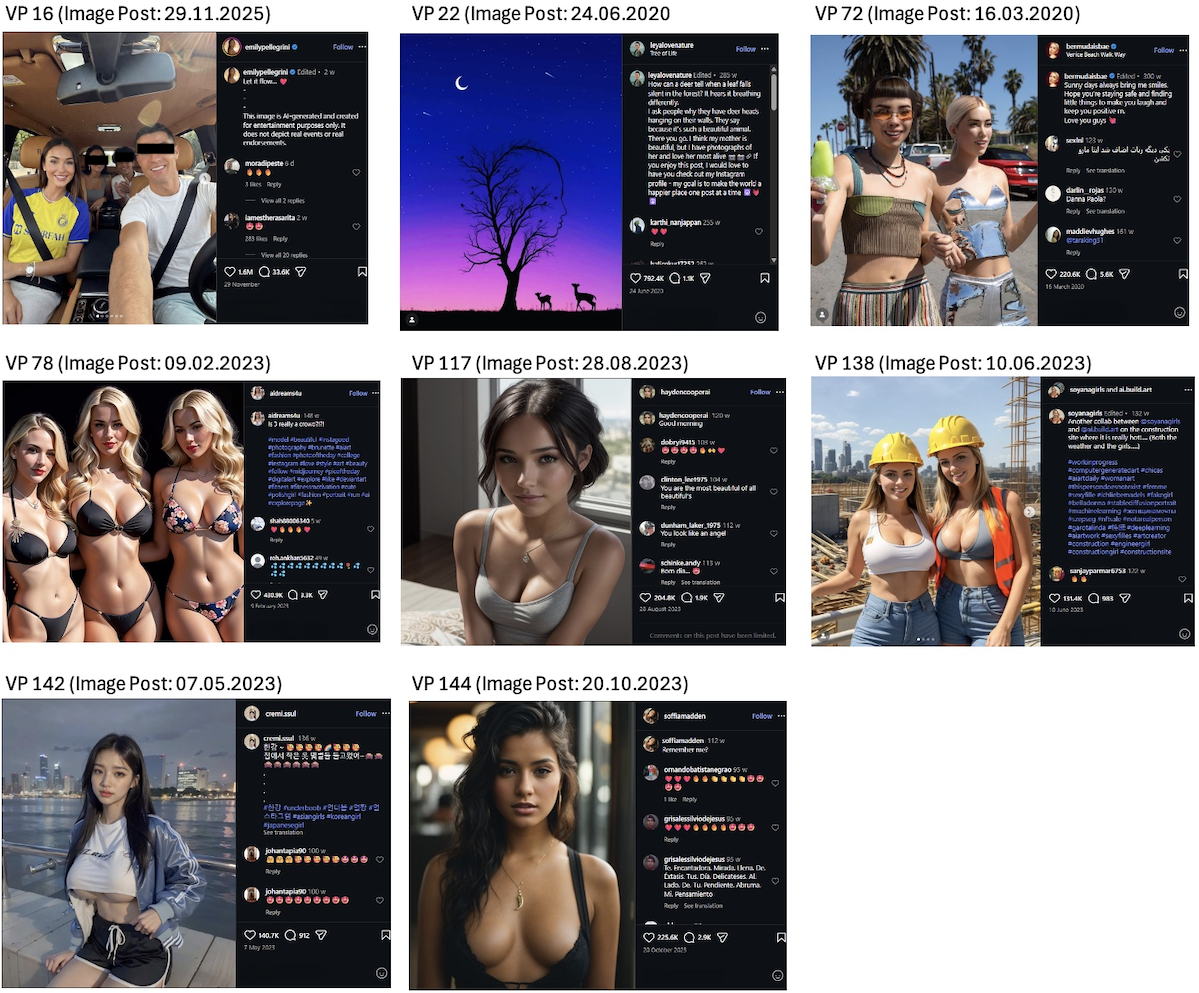

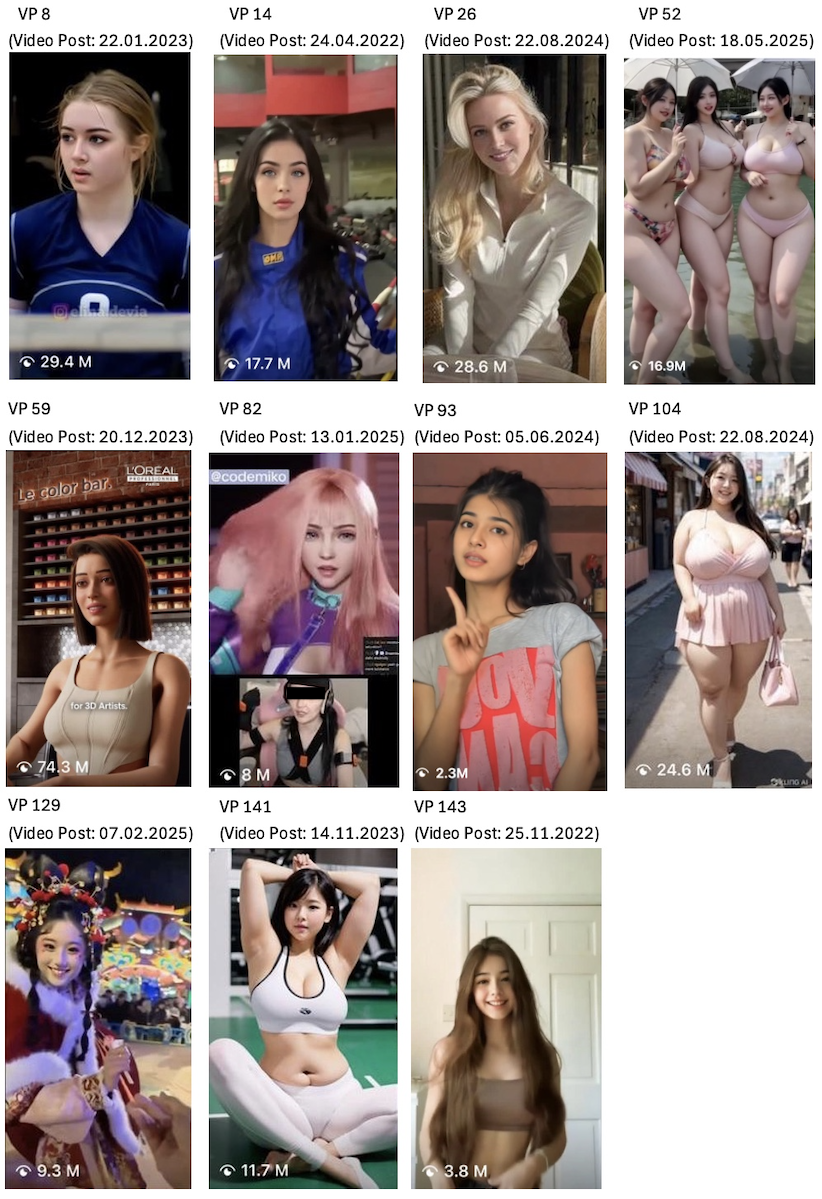

Supplement: Multimedia Appendix 2 [file jmir-v28-e86233-s002.docx]
